# Intra-abdominal pressure in severe acute pancreatitis

**DOI:** 10.1186/1749-7922-2-2

**Published:** 2007-01-17

**Authors:** Paivi Keskinen, Ari Leppaniemi, Ville Pettila, Anneli Piilonen, Esko Kemppainen, Marja Hynninen

**Affiliations:** 1 Department of Anesthesiology and Intensive Care Medicine, Meilahti Hospital, Helsinki University Central Hospital, PO Box 340, 00029 HUS, Helsinki, Finland; 2 Department of Gastroenterological and General Surgery, Meilahti Hospital, Helsinki University Central Hospital, Helsinki, Finland; 3 Department of Radiology, Meilahti Hospital, Helsinki University Central Hospital, Helsinki, Finland

## Abstract

**Background:**

Hospital mortality in patients with severe acute pancreatitis (SAP) remains high. Some of these patients develop increased intra-abdominal pressure (IAP) which may contribute to organ dysfunction. The aims of this study were to evaluate the frequency of increased IAP in patients with SAP and to assess the development of organ dysfunction and factors associated with high IAP.

**Methods:**

During 2001–2003 a total of 59 patients with severe acute pancreatitis were treated in the intensive care unit (ICU) of Helsinki University Hospital. IAP was measured by the intravesical route in 37 patients with SAP. Data from these patients were retrospectively reviewed.

**Results:**

Maximal IAP, APACHE II score, maximal SOFA score, maximal creatinine, age and maximal lactate were significantly higher in nonsurvivors. There was a significant correlation of the maximal IAP with the maximal SOFA, APACHE II, maximal creatinine, maximal lactate, base deficit and ICU length of stay. Patients were divided into quartiles according to the maximal IAP. Maximal IAP was 7–14, 15–18, 19–24 and 25–33 mmHg and the hospital mortality rate 10%, 12.5%, 22.2% and 50% in groups 1–4, respectively. A statistically significant difference was seen in the maximal SOFA, ICU length of stay, maximal creatinine and lactate values. The mean ICU-free days in groups 1–4 were 45.7, 38.8, 32.0 and 27.5 days, respectively. The difference between groups 1 and 4 was statistically significant.

**Conclusion:**

In patients with SAP, increased IAP is associated with development of early organ failure reflected in increased mortality and fewer ICU-free days. Frequent measurement of IAP during intensive care is important in optimizing abdominal perfusion pressure and recognizing patients potentially benefitting from decompressive laparotomy.

## Background

In spite of advances in the treatment of severe acute pancreatitis, the hospital mortality rate remains high [1,2]. The major determinants of death are multiple organ failure (MOF), the extent of necrotic pancreatic parenchyma and the presence of bacterial infection [3]. Recent clinical experience has indicated that some patients dying of "early MOF" might have suffered from untreated abdominal compartment syndrome (ACS). Massive fluid resuscitation in the early course of the disease combined with the severe inflammatory process in the retroperitoneum could contribute to visceral oedema leading to increased intra-abdominal pressure (IAP). This acute increase of IAP may in the severe cases lead to early organ dysfunction and ACS [4].

In healthy individuals, IAP ranges from 0 to 5 mmHg and varies with the respiratory cycle [5,6]. Because the organs and other contents in abdomen are relatively noncompressible, any increase in the volume of the retroperitoneal or abdominal contents increases IAP. New consensus definitions for IAH (intra-abdominal hypertension) and ACS were set by the World Society on the Abdominal Compartment Syndrome (WSACS) [7]. IAH is a sustained increase in IAP above 12 mmHg. ACS is a sustained increase in IAP above 20 mmHg with new onset organ failure with or without a low APP. However, even values of lower than 15 mmHg may cause organ dysfunction [8,9].

The prevalence of IAH in critically ill patients depends on the threshold used. Most commonly, the maximal IAP value instead of median or mean IAP has been used. In patients with severe acute pancreatitis, IAP >25 mmHg was detected in 30% of patients [10]. In a recent study, IAP > 15 mmHg was found in 78% of the patients with severe acute pancreatitis [11]. In a mixed population of ICU patients, prevalence of IAH (cut-off 12 mmHg) was 59%, 8.2% of these patients were classified as having ACS [12]. In another study with mixed ICU population, 32% of the patients had IAH (cut-off 12 mmHg) and 4.2% had ACS on admission [13]. In abdominal surgical patients, incidence of IAH (cut-off 20 mmHg) was 33 to 39% [14,15] and incidence of ACS (cut-off 20–25 mmHg) 2–36% [15-18].

This was a study on primary IAH and ACS according to the WSACS definitions. The aims of this study were to evaluate the degree of increased intra-abdominal pressure (IAP) in a group of patients with severe acute pancreatitis and to assess the development and progression of organ dysfunction and other factors associated with high IAP.

## Methods

### Patients

ICU computerized database was used to identify all patients with severe acute pancreatitis treated in the ICU of Helsinki University Hospital during 2001–2003. Severe acute pancreatitis was diagnosed, if the patients presented with abdominal pain, increased serum amylases and at least one organ dysfunction. The diagnosis was confirmed by abdominal CT in all but one patient. IAP was measured by the intravesical route in 37 of these 59 patients. IAP was measured if IAH was clinically suspected due to severe distension of the abdomen combined with a new or a worsening organ failure.

### Definitions

Acute Physiology And Chronic Health Evaluation (APACHE) II score [19] and Balthazar classifications [20] were used to assess the severity of pancreatitis. Balthazar classification: Grade A – normal CT, Grade B – focal or diffuse enlargement of the pancreas, Grade C – pancreatic gland abnormalities and peripancreatic inflammation, Grade D – fluid collection in a single location, Grade E – two or more collections and/or gas bubbles in or adjacent to pancreas. Sequential Organ Failure Assessment (SOFA) score [21] was calculated daily to assess the extent of organ dysfunction, using the worst value of each day. Based on the highest measured IAP value, the patients were divided into quartiles.

### Data collection

Patient data were retrospectively retrieved from the computerized patient database and the patient records. Age, gender, height, weight, body mass index (BMI), length of ICU stay, ICU-free days (out of 60 days), length of hospital stay, hospital mortality, medical history, etiology of pancreatitis and type of admission (primary or referral) were recorded. Parameters collected (days 1–14 in ICU) were IAP, lactate, C-reactive protein (CRP) at admission, creatinine, base deficit, need of renal replacement therapy, amount of fluids given, fluid balance, amount of peritoneal fluid (small, moderate or large; evaluated with CT), daily SOFA score, APACHE II score at admission and laparotomies during the ICU stay.

### Intra-abdominal pressure

Intra-abdominal pressure was measured through a Foley bladder catheter [22,23]. Intravenous infusion set was connected to normal saline, three-way stopcock and a disposable pressure transducer. The urometer was cut near the Foley catheter and the transducer was connected to the urometer by a three-way stopcock. The infusion set was flushed with saline and the pressure transducer was zeroed at the level of symphysis pubis. With the patient in supine position, 50 ml of saline were injected into the bladder and IAP was measured during end expiration. Nowadays minimal instillation volumes (<25 ml) are used for IAP measurement [7,24,25].

### Statistical analysis

Statistical analyses were performed using the SPSS software (Statistical package for the social sciences version 12.01, Chicago, IL, USA). Correlations between parameters were tested with Spearman's non-parametric correlation. Patients were divided into quartiles according to the maximal IAP measured. Kruskall-Wallis test was used to test statistical significance between multiple groups in continuous variables. Fisher exact test was used in dichotomous variables. Mann-Whitney test was used to test significance between two groups. Results are expressed as medians with interquartile (IQ) ranges. ICU-free days out of 60 are expressed as means. A p-value less than 0.05 was considered statistically significant.

## Results

The overall hospital mortality rate was 24% (9 out of 37) in our study group. According to the WSACS definition, IAH was found in 84% (31 out of 37) of patients. 17/37 (46%) patients had recurrent IAH. 18/37 (49%) patients had ACS and 7/37 (19%) had recurrent ACS. Table 1 summarizes the demographic and clinical data among survivors and nonsurvivors. The median age of the patients was 46 years (range 21–69 years), the most common etiological factor in this series was alcohol abuse (84%). Five patients underwent abdominal operations during the two first weeks in the ICU, additional four patients later on. The indication for two of the laparotomies during the study period was ACS. In patient 1 IAP decreased postoperatively from 33 mmHg to 16–22 mmHg. The urine output increased from less than 1 ml/kg/h to more than 2 ml/kg/h, plasma lactate normalized, and ventilatory function improved. In patient 2 IAP decreased postoperatively from 25 mmHg to 13 mmHg. The urine output increased from less than 0.5 ml/kg/h to 1.5 ml/kg/h and the ventilatory function improved. These effects on IAP were sustained in both of these patients over several days. Other reasons for laparotomy included intra-abdominal hemorrhage, suspected bowel perforation, and verified or suspected infection of the peripancreatic necrosis. 43% (16 of 37) of the patients required renal replacement therapy. For those 22 patients in whom the IAP was not measured the mortality rate was 18%.

**Table 1:** Demographic and clinical data of patients treated for severe acute pancreatitis.

	**All (%)**
	37 (100)
**Male**	33 (89)
**Age (years) median, range**	46 (21–69)
**BMI (kg/m2) median, range**	28 (21–42)
**Pre-existing diseases**	
Hypertension	10 (27)
Diabetes	2 (5)
Cardiovascular	3 (8)
Hyperlipidemy	2 (5)
Chronic pancreatitis	2 (5)
Respiratory	2 (5)
Renal	2 (5)
Psychiatric	4 (11)
**Etiology**	
Alcohol	31 (84)
Biliary	6 (16)
**Amount of peritoneal fluid in CT**	
small	13 (35)
moderate	19 (51)
large	4 (11)
**Balthazar classification**	
A, B	0 (0)
C	1 (3)
D	1 (3)
E	34 (92)
no CT	1 (3)
**Primary admission**	4 (11)

CT, computed tomography; BMI, body-mass index

**Figure 1:**
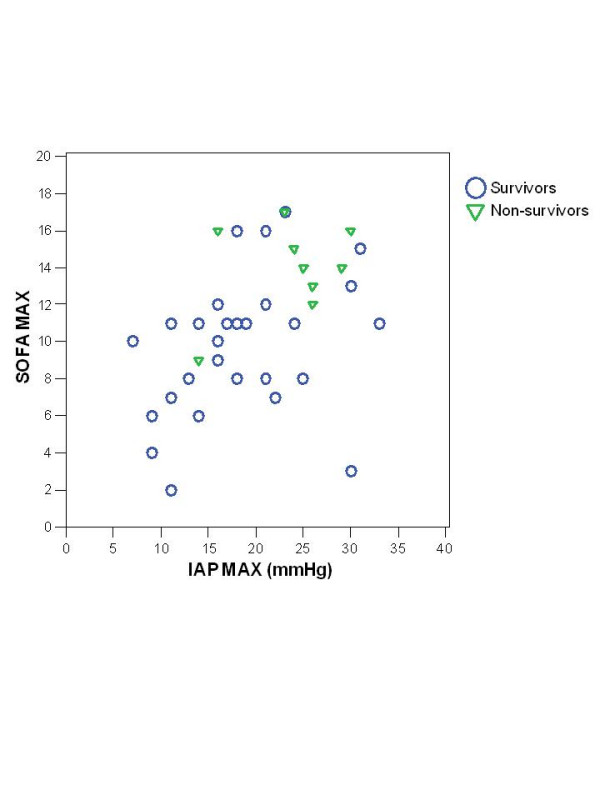
Correlation of maximal intra-abdominal pressure (IAP) with maximal Sequential Organ Failure Assessment (SOFA) score in survivors and nonsurvivors with severe acute pancreatitis.

The maximal IAP, APACHE II score at admission, maximal SOFA score, age, maximal plasma lactate, maximal creatinine and base deficit were significantly higher in the nonsurvivors (Table 2). There was a significant correlation of the maximal IAP with the maximal SOFA score (coefficient 0.49, p = 0.001) (Fig. 1), APACHE II score (0.50, p = 0.001), maximal lactate value (0.46, p = 0.002), base deficit (0.43, p = 0.008), maximal creatinine (0.56, p < 0.001) and duration of intensive care (0.48, p = 0.001). Maximal IAP did not correlate with the length of hospital stay or body-mass index (Spearman's non-parametric correlation).

**Table 2:** ICU data from survivors and nonsurvivors of severe acute pancreatitis.

	**Nonsurvivors**	**Survivors**	
	*Median (IQ range)*	*Median (IQ range)*	*p value*
**IAP max (mmHg)**	25 (19.5–27.5)	18 (13.3–22.8)	0.043
**SOFA max**	14 (12.5–16)	10.5 (7.3–11.8)	0.003
**ICU stay (days)**	27 (7.0–54.0)	15.5 (7.3–20.8)	0.257
**Hospital stay (days)**	28 (9.5–107.5)	26 (20.0–37.5)	0.986
**CRP at admission (mg/l)**	293 (212–385)	316 (246–378)	0.671
**APACHE II**	19 (17.0–22.5)	13 (10.0–17.0)	0.001
**Lactate max (mmol/l)**	2.7 (2.1–7.1)	1.5 (1.3–2.1)	0.006
**BE min (mmol/l)**	-10.5 (-13.2-(-8.0))	-1.3 (-6.6-(-1.3))	<0.001
**Creatinine max (mmol/l)**	338 (181.5–547)	140.5 (67.5–280.3)	0.020

IAP, Intra-abdominal pressure; SOFA, sequential organ failure assessment; BMI, body mass index; ICU, intensive care unit; CRP, C-reactive protein; APACHE II, Acure Physiology And Chronic Health Evaluation; BE, base excess

**Figure 2:**
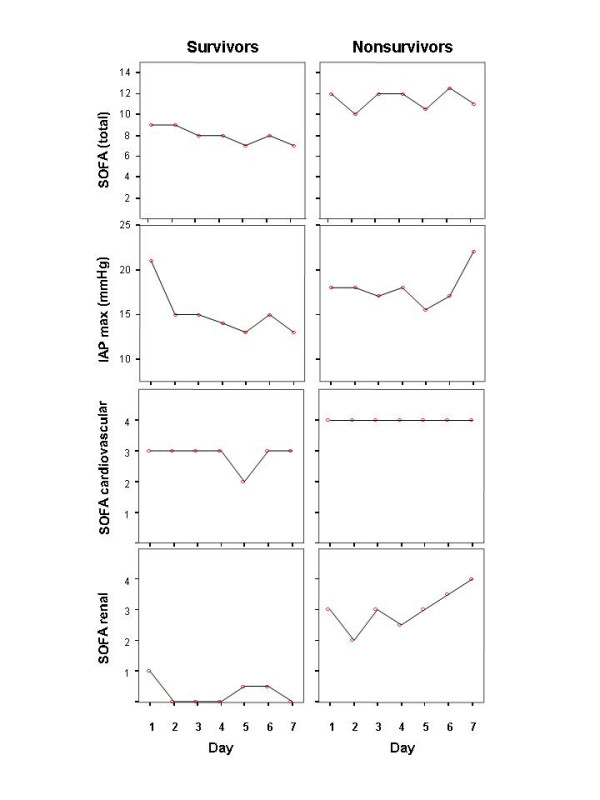
Maximal intra-abdominal pressure (IAP) values, total Sequential Organ Failure Assessment (SOFA) score, cardiovascular and renal SOFA scores during ICU-days 1–7 in the survivors and nonsurvivors of severe acute pancreatitis. Dots/lines represent medians.

**Figure 3:**
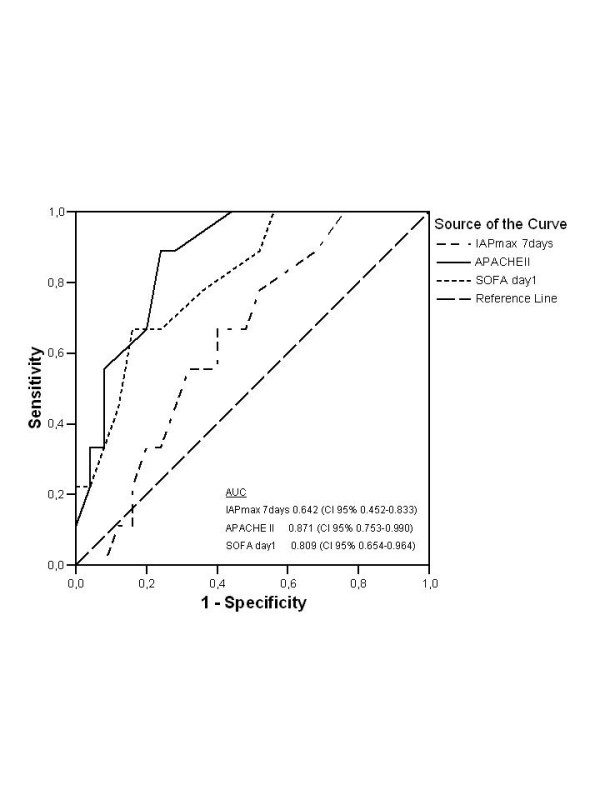
ROC curves for IAP max day 1–7, APACHE II and day 1 SOFA points. AUC: area under curve.

Figure 2 illustrates that nonsurvivors had higher maximal IAP, total SOFA, SOFA cardiovascular and renal scores on ICU-days 1–7 than survivors, whereas there was no significant difference in SOFA respiratory, coagulation, hepatic or neurological score (data not shown). Patients were divided into quartiles (8–10 patients in each group) according to the maximal IAP measured during days 1–14 in the ICU with maximum IAP values of 7–14, 16–18, 19–24 and 25–33 mmHg in groups 1 – 4, respectively. The hospital mortality rates in groups 1–4 were 10%, 12.5%, 22.2% and 50%, respectively. A statistically significant difference (Kruskall-Wallis test) between groups was seen in the maximal SOFA score (p = 0.01), maximal creatinine values (p = 0.01), duration of intensive care (p = 0.038) and maximal lactate values (p = 0.039). The difference in mortality rates between groups 1 and 4 was not statistically significant (p = 0.14; Fisher exact test). The mean ICU-free days in groups 1–4 were 45.7, 38.8, 32.0 and 27.5 days, respectively (p = 0.045, Kruskall-Wallis test). The difference in ICU-free days between groups 1 and 4 was statistically significant (p = 0.023, Mann-Whitney test). ROC (receiver operating characteristics) curves for IAP max day 1–7, SOFA day 1 and APACHE II are shown in Figure 3.

## Discussion

We found in this study, that high IAP in critically ill patients with acute pancreatitis correlates with the degree of organ dysfunction and length of intensive care. Increased IAP has deleterious effects on several organ systems. Cardiovascular effects include decrease in cardiac output, ventricular end-diastolic volume, preload and venous return, and increase in afterload and intrathoracic pressure [14,26]. Respiratory failure is caused by the elevation of the diaphragm leading to a decline in lung and chest wall compliance, decrease in functional residual capacity, total lung capacity and residual volume. Ventilation-perfusion mismatch leads to hypoxia, hypercapnia and need of mechanical ventilation. Renal dysfunction is probably caused by a decrease in renal perfusion pressure, the filtration gradient and renal blood flow. Splanchnic perfusion may diminish due to a decrease in cardiac output or a direct mechanical compression of the splanchnic bed. Increased concentrations of vasopressin may also play a role in the development of splanchnic ischemia [27-31]. Several scoring systems such as APACHE II [19] and SAPS [32] have been developed to predict outcome of critically ill patients. However, IAP is not included in any of these.

In earlier studies, an increase in IAP has been shown to be associated with increased mortality in surgical and trauma patients [17,18,29,30]. In trauma patients and liver recipients, acute ACS was associated with multiorgan failure and increased mortality [33-35]. In a recent multicenter study, the prevalence of IAH in critically ill patients was more than 50% [12]. In another study in a mixed ICU population, IAH during intensive care was an independent outcome predictor [13].

In our study, the hospital mortality rate showed an increasing trend from 10% to 50% with the maximal IAP increasing from 7–14 to 25–33 mmHg, respectively. The maximal IAP correlated with the highest SOFA score, APACHE II-score on admission, maximal lactate and creatinine values, base deficit and the duration of intensive care. For quartiles divided by maximal IAP, the mean ICU-free days significantly decreased with increasing maximal IAP values. In a recent study, where IAP was measured in patients with SAP only when IAH was clinically suspected, the incidence of IAH was 78%. This is in agreement with our study where the incidence of IAH was 84%. In the current study the IAP showed an increasing trend during the first week in the ICU in non-survivors, whereas it decreased in survivors during the same time period. In contrast, SOFA score remained relatively unchanged in non-survivors. This may indicate that IAP could be a sensitive indicator of poor prognosis in patients with SAP. However, a larger study to confirm this finding is needed.

As a limitation to this study IAP was not measured in all patients with SAP in our ICU during the study period. Also, the patient number was not large enough to compare the predictive value of different factors on patient outcome. However, the adverse effects of high IAP on different organ systems are fairly well documented and IAH may be a contributing factor to worsening organ function (SOFA score) in patients with SAP.

For patients with severe acute pancreatitis IAH could be especially deleterious because increased IAP in animal studies has been associated with bacterial translocation [36,37]. General splanchnic hypoperfusion and decreased blood flow to pancreas together with bacterial translocation may predispose the patient to infected necrosis and poor outcome [31]. However, the role and the treatment of IAH in severe acute pancreatitis still remains to be elucidated. Recently published international recommendations on the management of severe acute pancreatitis do not specifically address the management of IAH or ACS [38].

Once ACS is recognized, prompt treatment with decompressive laparotomy seems to be the best option although the exact indications, threshold IAP values and the most appropriate technique need further research. It is even more crucial in view of the considerable morbidity associated with the procedure itself, especially if the fascial closure is impossible leading to an open abdomen with significant long-term morbidity and need for reconstructive surgery of the abdominal wall later on. As shown in selected trauma and other surgical patients, however, the risk of organ dysfunction can be decreased with timely decompressive laparotomy in patients not responding to nonoperative management of severe IAH [18,39,40]. The same effect can be expected in patient with severe acute pancreatitis.

## Conclusion

In patients with severe acute pancreatitis, increased IAP is associated with development of early organ failure and fewer ICU-free days. Frequent measurement of IAP during intensive care in patients with severe acute pancreatitis could be important in optimizing abdominal perfusion pressure and recognizing patients potentially benefiting from early decompressive laparotomy.

## Competing interests

The authors declare that they have no competing interests.

## Authors' contributions

PK: Acquisition, analysis and interpretation of data, drafting the manuscript

AL: Interpretation of data, revising the manuscript critically

VP: Analysis and interpretation of data, revising the manuscript critically

AP: Analysis of data

EK: Revising the manuscript critically

MH: Analysis and interpretation of data, revising the manuscript critically

All authors read and approved the final manuscript
